# Sources of Social Support and Trauma Recovery: Evidence for Bidirectional Associations from a Recently Trauma-Exposed Community Sample

**DOI:** 10.3390/bs14040284

**Published:** 2024-03-29

**Authors:** Lauren M. Sippel, Rachel E. Liebman, Sarah K. Schäfer, Naomi Ennis, Alexandra C. Mattern, David C. Rozek, Candice M. Monson

**Affiliations:** 1 Department of Veterans Affairs Northeast Program Evaluation Center, West Haven, CT 06516, USA; 2 Department of Psychiatry, Geisel School of Medicine at Dartmouth, Hanover, NH 03755, USA; 3 National Center for PTSD, West Haven, CT 06516, USA; 4 Centre for Mental Health, University Health Network, Toronto, ON M5G 2C4, Canada; 5 Department of Psychiatry, University of Toronto, Toronto, ON M5T 1R8, Canada; 6 Department of Psychology, Toronto Metropolitan University, Toronto, ON M5B 2K3, Canada; 7 Leibniz Institute for Resilience Research, 55122 Mainz, Germany; 8 Technische Universität Braunschweig, 38106 Braunschweig, Germany; 9 VA Boston Healthcare System, Boston, MA 02130, USA; 10 Department of Psychiatry, Boston University Chobanian & Avedisian School of Medicine, Boston, MA 02118, USA; 11 Department of Psychology, The Pennsylvania State University, University Park, PA 16802, USA; 12 Department of Psychiatry & Behavioral Sciences, University of Texas Health Science Center, San Antonio, TX 78229, USA

**Keywords:** post-traumatic stress disorder, PTSD, social connectedness, emotional support, longitudinal, friends

## Abstract

Although the association between post-traumatic stress disorder (PTSD) and social support is well documented, few studies have tested the causal pathways explaining this association at several points in the acute post-trauma recovery period or examined whether the association varies for different sources of social support. To address these gaps, 151 community individuals (mean age = 37.20 years, 69.5% women) exposed to trauma within the previous 6 months were recruited to complete measures of PTSD and social support from intimate partners, friends, and relatives four times in 1 year. In line with recent recommendations for research on social support and PTSD symptoms, random intercept cross-lagged panel modeling (RI-CLPM) was used to examine dynamic changes between PTSD severity and social support over time. The pattern of RI-CLPM cross-lagged coefficients indicated that positive deviations from one’s expected stable level of total social support (across all sources) sped up the recovery of PTSD symptoms at the end of the post-trauma year, and more severe PTSD symptoms than expected based on one’s expected stable level of PTSD started eroding social support midway through the assessment year. When specific sources of social support were analyzed separately, the association between within-person increases in social support from friends at any given time point accelerated the recovery from PTSD across the entire year. Among participants with intimate partners (*n* = 53), intimate partner support did not predict PTSD symptoms, but more severe PTSD symptoms at any given time point predicted less support at the following time point. Results from this longitudinal study provide additional support for the bidirectional relationship between PTSD and social support over time and suggest that perceived social support from friends may be especially helpful during trauma recovery.

## 1. Introduction

Post-traumatic stress disorder (PTSD) is an often-debilitating disorder characterized by intrusive thoughts and memories, avoidance, hypervigilance, negative mood, and physiological arousal [1]. Between 75 and 90% of the general population is exposed to potentially traumatic events [2,3]. Most of these individuals experience some post-traumatic stress symptoms that naturally abate within the first few weeks post trauma [4,5]. However, approximately 8% of trauma-exposed individuals do not experience this “natural recovery” and instead go on to be diagnosed with PTSD [2]. Identifying factors that influence the risk of PTSD after exposure to trauma is critical for early intervention efforts.

Interpersonal factors are increasingly recognized as critical to the onset, maintenance, and treatment of PTSD [6,7,8]. Post-traumatic social support, in particular, is a well-documented risk and resilience factor for PTSD outcomes [9,10,11]. Several theories have been put forth to elucidate the causal links between PTSD and social support [8]. According to social causation theory [12] and the stress buffering model [13], social support contributes to the risk of PTSD after trauma, in that stronger social support can buffer against PTSD and poorer social support increases the risk of PTSD. The social erosion theory posits that PTSD symptoms degrade social support over time via their negative impact on relationships [12]. Evidence from prospective and meta-analytic studies supports both causal directions, indicating a bidirectional relationship [14,15,16].

This converging evidence of the bidirectional relationship between PTSD and social support is limited by the heterogeneity in assessment methods between studies [15,16]. Sources of heterogeneity include variation in the timeline of assessment protocols (e.g., how soon after trauma social support and PTSD symptoms are measured, the length of assessment windows) and the types and sources of social support studied. Social support is a complex and multifaceted construct that can be disentangled into different domains and functions [8,16]. Perceived emotional social support, defined here as the perception of one’s emotional needs being supported by close others [17], is most consistently associated with PTSD-related outcomes across studies [8,18,19], whereas meta-analytic findings show that negative social reactions have the strongest association with PTSD symptoms [16,20].

The value of social support for promoting trauma recovery may vary according to who is providing the support and when the support is provided [11]; similarly, PTSD symptoms may have a greater impact on support provided by some sources than others. In their meta-analyses, Zalta and colleagues (2021) found that the magnitude of the PTSD–social support association did not vary by support source after adjusting for covariates, whereas Wang and colleagues (2021) found that PTSD predicted greater reductions in support from close others (i.e., family members, friends) than from distant others like units or organizations. This inconsistency in findings for specific sources of social support may be due to different coding conventions for types of relationships and the fact that Wang’s meta-analysis was limited to longitudinal studies, whereas Zalta’s was not.

Most primary studies that have attempted to disentangle the effects of social support from specific sources have been cross-sectional studies conducted among military veterans, with inconsistent results. For example, among combat veterans, social support from intimate partners, family, and military peers (but not friends) negatively correlated with PTSD [21]. A study of veterans who served in Iraq and Afghanistan indicated that support from all sources (immediate family, other family, friends, coworkers, and community) was associated with lower odds of a positive PTSD screen [22]. Higher perceived support from family and friends was related to less severe PTSD but support from the general public was not [23]. Finally, in a study of male veterans being treated for chronic PTSD, more severe PTSD predicted erosion in support from nonveteran friends but not from relatives [24].

The timing of assessments of PTSD and social support, in terms of how soon after trauma they are administered and how frequently, vary across studies and the specific empirical questions being examined. Some have measured PTSD and social support over intervals of several months within the first 2 years post trauma [12,25,26]. Other studies have included relatively fewer time points in the more acute post-trauma period (e.g., days or weeks; [27]) or several years after trauma exposure [28,29]. Studies that incorporate frequent assessment of PTSD symptoms and social support across a relatively limited time frame soon after trauma may be best positioned to capture meaningful and dynamic changes in associations between perceived social support and PTSD severity during the acute post-trauma recovery period while minimizing the risk of capturing more chronic forms of psychopathology that may distort these associations.

To our knowledge, no longitudinal studies have examined the association between social support from multiple distinct sources of social support and PTSD symptoms in a community sample of adults soon after exposure to trauma. Such work has important implications for identifying interpersonal intervention targets in the acute trauma recovery period that are generalizable to different populations of trauma survivors.

### The Current Study

The objective of the current study was to examine the dynamic associations between PTSD and social support over time in a sample of individuals recruited from the community within 6 months of trauma exposure. Participants were assessed four times over a 12-month period to allow adequate time for the many trajectories of post-trauma recovery observed in the literature (e.g., natural recovery that is most likely within three months, delayed expression, etc.; [4,5]) to be represented and investigated in relation to social support. As presented in Monson et al., 2023 [30], a total of 44% of participants met the diagnostic criteria for PTSD at the first assessment; the rates of PTSD declined over time (23% at Time 2, 18% at Time 3, and 11% at Time 4). In the current study, we addressed previous criticisms of the designs of longitudinal studies of social support and PTSD symptoms [31] by using random intercept cross-lagged panel modeling (RI-CLPM) to test pathways between PTSD symptoms and total social support (i.e., across all types of relationships), followed by an examination of support from three types of close others (per Wang et al., 2021), intimate partners, relatives, and friends, using four time-points. In RI-CLPM, significant cross-lagged effects indicate the extent to which deviations from the trait level in one variable at a given time point can explain change in the other variable at the following time point. Throughout the remainder of the manuscript, we refer to such deviations from the trait level as “deviations from the expected stable level” to disambiguate PTSD and social support from being interpreted as “traits”. 

In RI-CLPM terminology, we hypothesized that one’s stable mean level of social support would be associated with one’s stable mean level of PTSD symptoms, with PTSD and social support being negatively associated. Moreover, we hypothesized reciprocal within-person dynamics over time, again with PTSD and social support being negatively associated. In other words, both on average and over time, we expected a negative association between PTSD and social support (i.e., greater social support associated with less severe PTSD symptoms or, vice versa, poorer social support associated with more severe PTSD symptoms). Based on our literature review, we hypothesized that these bidirectional associations would hold for all sources of social support but would be particularly strong for social support from intimate partners and friends compared to relatives. Lastly, we explored how these dynamics varied across the 12-month study period.

## 2. Materials and Methods

### 2.1. Participants

The current study was derived from a larger longitudinal study examining risk and resilience factors for PTSD among a sample of 151 recently traumatized adults [30]. To be eligible for participation, participants had to be: (a) between 18 and 75 years old; (b) exposed to a DSM-IV-TR Criterion A trauma within six months prior to study enrolment; and (c) fluent in English. Sample demographics are presented in Table 1 and described in detail in [30]. Briefly, the majority of participants self-identified as women (69.5%), single (51.7%), unemployed (54.3%), and had an annual income less than CAD 35,000 per year (71.6%). The mean age of the sample was 37.20 years (SD = 14.05; men M = 39.91, women M = 36.07). The most commonly identified race/ethnicity was white (47.7%). The majority of participants reported directly experiencing the traumatic event, and the most commonly experienced traumatic events were accidents (30.5%), sexual assaults (21.9%), and physical assaults (21.2%). The average time elapsed between trauma exposure and the initial assessment was 127.9 days (SD = 56.6; i.e., approximately four months).

### 2.2. Procedure

This study was approved by the Research Ethics Board at Ryerson University (now Toronto Metropolitan University). Participants were recruited from the Toronto, Canada area using clinician referrals, newspapers, online advertisements, and flyers posted at university campuses, community centers, religious centers, and hospitals with trauma centers. Following informed consent, eligible participants completed four identical assessments that consisted of a clinician-administered interview and self-report questionnaires. A full description of the study procedures is provided in the primary outcomes paper [30].

### 2.3. Measures

PTSD: As this study commenced prior to the publication of the *Diagnostic and Statistical Manual of Mental Disorders, Fifth Edition* [32], the Clinician-Administered PTSD Scale, Fifth Edition [33] was not yet available. Thus, the current study used the Clinician-Administered PTSD Scale (CAPS; [34]), a semi-structured clinical interview that assesses for DSM-IV-TR PTSD diagnostic criteria and symptom severity. In an effort to account for anticipated changes to the DSM, items that correspond to anticipated DSM-5 diagnostic criteria for PTSD were also included in the version of the CAPS used in this study. PTSD symptom presence and severity were assessed over the past month. The frequency and intensity of the 20 symptoms comprising the PTSD diagnosis in the DSM-5 were each rated on a 5-point scale, consistent with the original CAPS [34]. Higher scores are indicative of greater symptom severity.

For the current data set, the internal reliability of the CAPS was excellent at each assessment point (Cronbach’s α’s = 0.94 to 0.97). To test interrater reliability, a random sample (8.5%) of all interviews conducted was selected for independent expert assessment monitoring. The intraclass correlation coefficient (ICC) for these ratings was excellent (ICC = 0.99).

Social support: The Provisions of Social Relations Scale (PSRS; 17) is a 22-item measure for assessing individuals’ perceptions of the availability of social support from different potential support providers, including intimate partners, friends, and relatives. The PSRS is composed of three subscales: Intimate Partner Support (PSRS Partner; six items), Relatives Support (PSRS Relatives; eight items), and Friend Support (PSRS Friends; eight items). Each subscale assesses the degree to which the respondent feels close to the target support provider, perceives the support provider as willing to take the time to talk and have communication of worth, has confidence that the support source will be there when needed, and has the belief that the support source has confidence in the respondent. Respondents rate their agreement using a 4-point Likert scale ranging from 1 (very much like my experience) to 4 (not at all like my experience). In the current study, the scale was reverse-scored for data analytic purposes so that higher scores reflected higher levels of perceived social support.

All participants were administered the Perceived Family Support and Perceived Friend Support scales. Respondents who did not have an intimate partner did not complete the Perceived Partner Support scale. A total score was calculated using the mean of the appropriate scales [17]. Internal consistency was high for each subscale across time points (Cronbach’s α’s = 0.90–0.97).

### 2.4. Analytic Plan

We tested a random intercept cross-lagged panel model (RI-CLPM; [35]), a type of structural equation model (SEM), to examine the stability and reciprocal relationships of PTSD and social support over time. A benefit of the RI-CLPM over the traditional cross-lagged panel model (CLPM) is its ability to separate within-person variations from stable, between-person (i.e., trait-like) variations by including separate time-invariant random intercept factors that account for between-person mean-level differences in the outcome of interest. Therefore, in contrast to the traditional CLPM, the RI-CLPM estimates both stable differences between people as well as within-person variations within (autoregressive) and between (cross-lagged) measures over time [35]. In the context of the RI-CLPM model, autoregressive change refers to a temporary deviation from the stable trait level in Construct X at time t predicting a change in Construct X at time t + 1, and cross-lagged change refers to a temporary deviation from the stable trait level in Construct X at time t predicting a change in Construct Y at time t + 1.

In the current study, autoregressive and cross-lagged paths between PTSD symptoms and social support were examined across the four assessment intervals. Positive autoregressive effects denote that a stronger positive deviation from one’s stable level of PTSD symptoms or social support is associated with a subsequent increase in the respective measure. Cross-lagged paths were explored to examine bi-directional relationships between PTSD symptoms and social support over time, with significant cross-lagged effects indicating the extent to which deviations from the trait level in one variable at a given time point can explain change in the other variable at the following time point. As previously noted, we refer to such deviations from the trait level as “deviations from the expected stable level” to disambiguate PTSD and social support from being interpreted as “traits”. Analyses were conducted with full information maximum likelihood (FIML) estimation to account for missing data. FIML uses all available observed data to estimate coefficients and is robust to biased estimates that can occur when data are not missing completely at random [36].

Descriptive analyses were conducted in SPSS version 25.0 (IBM, 2018). RI-CLPM analyses were conducted using the Mplus statistical package (Version 7; [37]). A total of four RI-CLPMs were conducted between PTSD and each of the social support subscales (total, friends, family, intimate partners). To examine if autoregressive and cross-lagged associations varied across time, we tested the longitudinal invariance of the autoregressive and cross-lagged paths in three models: (1) all autoregressive and cross-lagged paths fixed across time, (2) autoregressive paths freely estimated and cross-lagged paths fixed across time, and (3) all parameters freely estimated. To determine the model with the best fit, each model was compared against the most saturated model (i.e., the freely estimated model) on the Chi-square goodness of fit test (χ^2^), the Comparative Fit Index (CFI; [38]), the Root Mean Square Error of Approximation (RMSEA; [39]) and the Akaike Information Criterion (AIC; [40]) to determine the best fitting model. CFI values greater than 0.95 indicate good model fit [41]. RMSEA values of 0.01 and 0.05 represent excellent and good fit, respectively [42]. Smaller AIC values indicate a better fitting model when comparing competing models [40,41]. The fit was considered to be significantly better if the Chi-square difference was significant, the change in CFI was <0.01, and the change in RMSEA was <0.015 [43].

## 3. Results

Means and standard deviations for PTSD symptoms and social support subscales at each assessment point can be found in Table 2. Bivariate correlations for PTSD symptoms and social support subscales at each assessment point are found in Supplemental Table S2. PTSD symptoms and overall social support were negatively correlated at all time points (r ranging from −0.16 to −0.42).

Table 3 shows model fit estimates and results of Chi-square difference tests for each model. For the association between PTSD severity and total social support, the model in which all paths were freely estimated demonstrated the best fit, indicating that autoregressive and cross-lagged associations varied over time. For the three other models, freely estimated autoregressive paths and constrained cross-lagged paths demonstrated the best fit, suggesting that the autoregressive associations of each construct varied over time, but the cross-lagged associations between them were stable.

### Bidirectional Longitudinal Associations between PTSD Symptoms and Social Support

**Figure 1:**
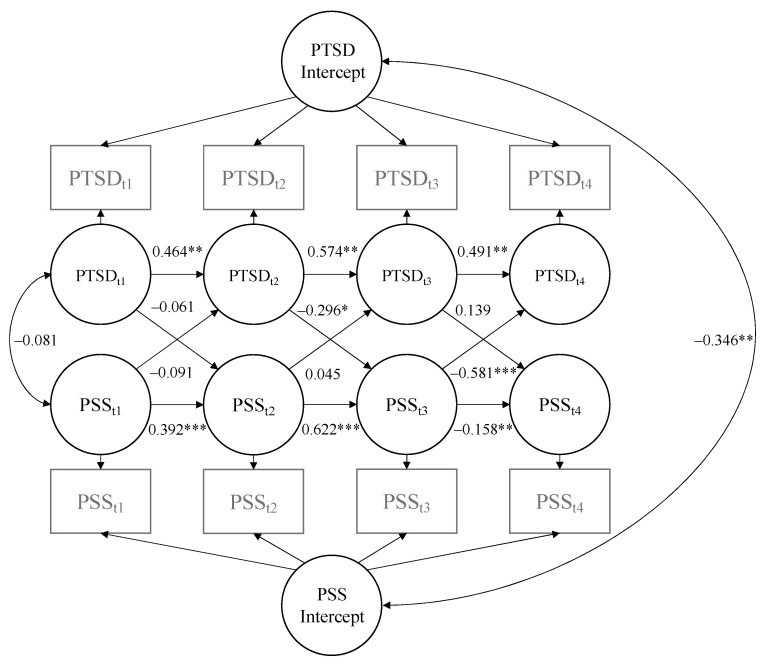
Random intercept cross-lagged panel model illustrating autoregressive and cross-lagged coefficients for PTSD and total social support. PSS = perceived social support; PTSD = post-traumatic stress disorder. * *p* < 0.05. ** *p* < 0.01. *** *p* < 0.001.

PTSD symptoms and total social support: As shown in Table 3, the fit for the bidirectional model between total PTSD severity and total social support was good. As displayed in Table 4 and Figure 1, there was a significant and negative correlation between the random intercepts of PTSD symptoms and total social support, indicating that in terms of stable between-participant differences, higher total social support was associated with less severe stable PTSD symptoms. There were significant and positive autoregressive effects for PTSD symptoms at all three time points, as well as for total social support at time points 1 and 2. There were also significant and negative cross-lagged effects of total social support on PTSD symptoms from time 3 to time 4, and of PTSD on total social support from time 2 to time 3. This pattern of cross-lagged coefficients suggests that having more severe PTSD symptoms than expected based on one’s stable level of PTSD erodes social support midway through the assessment year and that having more social support than expected based on one’s stable level of social support promotes the recovery of PTSD symptoms at the end of the post-trauma year. 

PTSD symptoms and relative social support: The fit for this bidirectional model was also good (see Table 3). As shown in Table 4, there was a significant and negative correlation between the random intercepts of PTSD severity and social support from relatives, such that higher stable levels of social support from relatives were associated with less severe stable PTSD symptoms. There were significant and positive autoregressive effects for PTSD at time 1, as well as for relative social support at time 2. There were no significant cross-lagged effects of PTSD on relative social support or vice versa.

PTSD symptoms and friend social support: The fit for this bidirectional model was likewise good (see Table 3). There was not a significant correlation between the random intercepts of PTSD severity and social support from friends, such that higher stable social support from friends was unrelated to stable PTSD symptom severity (see Table 4). There were significant and positive autoregressive effects for PTSD at time 3, as well as for friend social support at time points 1 and 2. There were significant and negative cross-lagged effects of social support from friends on changes in PTSD severity, as well as of total PTSD severity on change in social support from friends over all three assessment intervals. This pattern of cross-lagged coefficients suggests that more social support from friends than expected based on one’s stable level speeds up the recovery from PTSD and that more severe PTSD symptoms than expected based on one’s stable level of PTSD predicts decreasing social support from friends over the entire year post traumatization.

PTSD symptoms and intimate partner social support: The fit for these bidirectional models was also good (see Table 3). As shown in Table 4, there was not a significant correlation between the random intercepts of PTSD and social support from intimate partners; one’s stable level of intimate partner social support was not associated with one’s stable level of PTSD symptoms. There were no significant autoregressive effects for PTSD symptoms across the assessment year. There was a significant and positive autoregressive effect of intimate partner social support at time 2. There were no significant cross-lagged effects of intimate partner support on PTSD symptoms, but there were significant and negative cross-lagged effects of PTSD symptoms on intimate partner social support, indicating that intimate partner social support does not impact PTSD but that higher-than-expected (based on one’s stable level) PTSD symptoms at any given time point do predict decreased social support from intimate partners at the following time point.

## 4. Discussion

The association between PTSD and social support is well established, but there remains a lack of clarity about the nature of this association at different points in the post-trauma period and whether it varies according to the source of social support. Given the lack of longitudinal data in this area, we examined both PTSD symptoms and perceived social support from multiple distinct support sources in a community sample of adults after exposure to trauma over time. In line with previous studies [15,16], social support and PTSD symptoms were negatively associated (i.e., higher stable levels of total social support were associated with less severe PTSD symptoms and lower levels of support were associated with more severe PTSD symptoms). However, beyond previous studies, we found evidence for complex within-participant dynamics, pointing to a bidirectional relationship between PTSD severity and total social support. Namely, more severe PTSD symptoms than expected based on one’s stable level eroded subsequent total social support midway through the assessment year, while having more total social support from all sources than expected based on one’s stable level sped up the reduction in PTSD symptoms at the end of the post-trauma year.

In terms of specific sources of support, the pattern of findings was similar for support from friends, except that the bidirectional relationship between PTSD severity and support from friends was stable throughout the entire post-trauma year. Intimate partner support did not predict PTSD severity, but more severe PTSD symptoms than expected based on one’s stable level of PTSD predicted less support from intimate partners. Most extant studies of PTSD and social support from specific sources have been cross-sectional studies with military veterans [21,22,23]. In these studies, the effect sizes for the PTSD–social support association were larger than the effect sizes observed among civilians [15,16]. Our longitudinal research replicates and extends the previous findings in veterans, suggesting that promoting social support in the post-trauma period is critical to trauma recovery across populations.

Our findings largely align with those of two recent meta-analyses of PTSD and social support [15,16] that documented a bidirectional relationship between PTSD and social support. With respect to total social support, our finding that total social support predicted PTSD is in line with social causation and stress buffering at the end of the assessment year. The finding that PTSD predicted social support is in line with social erosion mid-way through the year. We found that this association differed across sources of support, with support from friends especially salient in both causal directions, and PTSD predicting reductions in support from intimate partners. These findings are consistent with Wang and colleagues’ conclusion and interpersonal conceptualizations of PTSD that posit that PTSD degrades support from close others such as friends and intimate partners [6], perhaps via PTSD’s negative effects on emotional closeness, reactivity, and disclosure, all of which are highly salient to the quality of close relationships. This important buffering effect of support from friends on psychiatric outcomes has also been observed in other populations, such as university students, for whom social support from friends reduced depressive symptoms [44]. Taken together, these findings suggest that friends may be an under-appreciated but promising source of social support in the post-trauma period because of the inherent value of them being intentionally selected and maintained as close others. Unfortunately, this may not be assumed for relatives and even intimate partners, with whom relationships may be sustained even if quality is compromised. Successful, high-quality friendships may be especially valuable for reducing stress and enhancing positive affect and quality of life.

These findings support the early intervention technique of building and leveraging social support in the early post-trauma recovery period, as well as mitigating the negative effects of PTSD on social support and close relationship quality once PTSD is present. Dyadic and systemic approaches to PTSD intervention have promising evidence for improving both PTSD symptoms and relationship quality (e.g., Cognitive Behavioral Conjoint Therapy for PTSD; [45,46]). Educating loved ones about PTSD symptoms and treatment [47] may serve as a lighter-touch approach for enhancing loved ones’ skills in providing emotional support to patients while they engage in PTSD treatment. As suggested by our findings, friends may serve as especially potent sources of support and could benefit from guidance regarding how and when to provide social support that is best matched to the trauma-exposed individuals’ needs. For example, friends of trauma-exposed individuals could be encouraged to promote trauma recovery by providing positive support (e.g., emotional validation, promoting thoughts and feelings of safety) and avoiding negative reactions such as treating the survivor differently, rejection, and blaming—reactions that have particularly detrimental effects on trauma recovery [16,20,48]. Engaging with supportive others could also promote cognitive changes, in that they could gently challenge overly negative beliefs and offer alternative interpretations, which in turn can facilitate trauma recovery [49]. From a public health perspective, as friendship networks tend to degrade in later age [50], it may be especially important to instill and encourage recognition of the value of friendships early in life as a strategy for enhancing resilience to stress and promoting overall health and wellness [51].

This study has several limitations. Although our study addressed previous criticisms of longitudinal studies of social support and PTSD symptoms by including four waves and using RI-CLPM [31,35], our statistical models were complex and statistical power was not adequate to go beyond our key questions to test moderators of this association (e.g., trauma type, gender), add covariates to the model (e.g., time since trauma, other forms of psychopathology that may contribute to perception of social support like depression), or directly compare sources of social support. Gender differences in the PTSD–social support association may be particularly interesting to examine given that women are at double the risk of PTSD than men [3], that patterns of trauma exposure and post-trauma psychopathology differ for men and women (e.g., with depression more common among women and substance use disorders more common among men [52]), and that women have been found to exhibit affiliative responses to stress [53], suggesting that women may be especially likely to benefit from social support after trauma. Future research could also examine the interplay between sex and gender differences and different types of trauma in relation to social support, as well as sex and gender differences in the likelihood of providing support to trauma-exposed family and friends. We were also not able to account for co-occurring mental health symptoms that could affect perceptions of social support, such as depression. The analysis of social support from intimate partners, in which we found that PTSD predicted reductions in support from intimate partners, should be interpreted with caution, as it was limited by only about one third of the sample completing that subscale. Future research that is specifically designed and powered to examine social support from intimate partners can inform whether this form of support is differentially protective or negatively impacted by PTSD relative to support from other sources, as well as how it can be optimally improved via early intervention.

## 5. Conclusions

Overall, results from this longitudinal study provide additional support for the bidirectional relationship between PTSD and social support over time in a recently trauma-exposed community sample and suggest that perceived social support from friends may be especially helpful during trauma recovery. Future studies could examine the quality of social support relationships in terms of disentangling positive and negative reactions to trauma and emotional disclosures, as well as more closely controlling for the time since the trauma to delineate the directionality of the association between PTSD symptoms and social support in pre-specified post-trauma recovery periods. This will ultimately improve the health and recovery trajectory of the many people who are affected by trauma and those who surround them.

## Figures and Tables

**Table 1:** Demographic and clinical information for the sample at initial assessment (N = 151).

Variable	*n* (%)
Self-identified gender ^a^	
Men	43 (28.5)
Women	105 (69.5)
Ethnicity ^b,c^	
Aboriginal	6 (4.0)
Black	21 (13.9)
South, West, South East, East Asian	30 (19.8)
White	72 (47.7)
Mixed or Other Ethnicity ^d^	18 (11.8)
Marital Status	
Single	78 (51.7)
Committed Relationship/Married	42 (27.8)
Divorced, Separated, Widowed	27 (17.9)
Employed	69 (45.7)
Annual Income (CAD)	
<5000	30 (19.9)
5000 to 14,999	37 (24.5)
15,000 to 34,999	41 (27.2)
35,000 to 49,999	19 (12.6)
>50,000	15 (9.9)
Type of Traumatic Event based on CAPS Interview	
Sexual Assault	33 (21.9)
Physical Assault	32 (21.2)
Accident	46 (30.5)
Sudden Illness or Death	21 (13.9)
Robbery/Home Invasion	4 (2.6)
Threatened by Other	5 (3.3)
Other Trauma Type ^e^	8 (5.3)

^a^ Missing *n =* 3. ^b^ Missing *n =* 4. ^c^ See Supplemental Table S1 for age and gender breakdown by racial/ethnic group. ^d^ Other ethnicity included Latin American and Caribbean. ^e^ Other trauma includes being held hostage, dog attacks, witnessing attempted suicide, and natural disasters. CAD = Canadian dollar.

**Table 2:** Means and standard deviations for outcome variables across assessment points.

	Time 1			Time 2			Time 3			Time 4		
	*n*	*M*	*SD*	*n*	*M*	*SD*	*n*	*M*	*SD*	*n*	*M*	*SD*
CAPS	149	53.66	31.70	122	38.65	32.56	118	29.00	30.73	123	24.97	31.05
PSRS Total	142	20.73	6.14	116	21.05	5.67	112	20.95	5.66	116	20.77	5.68
PSRS Relatives	143	19.68	7.76	117	19.61	7.61	113	19.51	7.41	117	19.59	17.88
PSRS Friends	143	21.98	7.25	116	22.9	6.52	112	22.85	6.63	119	22.64	6.55
PSRS Partner	53	18.89	5.25	43	20.19	4.16	40	19.88	4.44	49	20.61	4.11

Note. CAPS = Clinician-Administered PTSD Scale for DSM-IV [34]; PSRS = Provision of Social Relations Scale [17].

**Table 3:** Model fit estimates for the random intercept cross-lagged panel model.

Model	df	χ^2^	*p*	RMSEA	CFI	AIC	χ^2^ Diff *p*
CAPS + PSRS Total							
All paths fixed	17	52.27	0.00	0.12	0.96	7340.16	0.00
Only cross lags fixed	13	26.44	0.01	0.08	0.98	7322.34	00.01
All paths free	9	12.95	0.16	0.05	0.995	7316.85	
CAPS + PSRS Relatives							
All paths fixed	17	44.11	0.00	0.10	0.97	7620.20	0.00
Only cross lags fixed	13	22.90	0.04	0.07	0.99	7606.98	0.11
All paths free	9	15.46	0.08	0.07	0.99	7607.54	
CAPS + PSRS Friends							
All paths fixed	17	71.89	0.00	0.15	0.93	7605.10	0.00
Only cross lags fixed	13	25.54	0.01	0.08	0.98	7566.75	0.11
All paths free	9	18.03	0.03	0.08	0.99	7567.25	
CAPS + PSRS Intimate Partner							
All paths fixed ^a^	18	47.77	0.00	0.11	0.95	5593.05	0.00
Only cross lags fixed	13	31.12	0.00	0.10	0.97	5586.39	0.11
All paths free ^b^	10	25.09	0.01	0.10	0.97	5586.36	

Note: Models were compared with Chi-square difference tests. ^a^ To facilitate convergence covariance between time 2 PTSD and social support set to 0. ^b^ To facilitate convergence covariance between time 4 PTSD and social support set to 0. CAPS = Clinician-Administered PTSD Scale [34]. PSRS = Provisions of Social Relations Scale [17].

**Table 4:** Model fit indices and standardized parameter estimates for clinician-rated PTSD with social support random intercept cross-lagged panel model.

	Total PSS		Relative PSS		Friend PSS		Intimate Partner PSS	
	Est	SE	Est	SE	Est	SE	Est	SE
Autoregressive Coefficients								
PTSD_1–2_	0.464 **	0.149	0.421 ***	0.092	0.277 *	0.137	0.317 *	0.150
PTSD_2–3_	0.574 **	0.175	−0.384	0.245	0.286	0.177	0.293	0.303
PTSD_3–4_	0.491 *	0.209	−561*	0.285	0.484 **	0.108	0.380	0.238
PSS_1–2_	0.392 ***	0.107	0.274	0.212	440 **	0.131	0.197	0.258
PSS_2–3_	0.622 ***	0.113	0.838 ***	0.068	0.809 ***	0.062	0.905 ***	0.067
PSS_3–4_	−0.158	0.408	0.343	0.216	0.219	0.248	0.217	0.347
Cross-Lagged Coefficients								
PSS_1_→PTSD_2_	−0.091	0.124	0.142	0.099	−0.344 *	0.177	−0.349	0.175
PSS_2_→PTSD_3_	0.045	0.205	0.334	0.222	−0.314 *	0.126	−0.288	0.153
PSS_3_→PTSD_4_	−0.581 ***	0.151	0.155	0.109	−0.305 *	0.109	−0.320	0.169
PTSD_1_→PSS_2_	−0.061	0.148	−0.047	0.080	−0.181 *	0.076	−0.180 *	0.090
PTSD_2_→PSS_23_	−0.296 *	0.115	−0.035	0.059	−0.158 *	0.071	−0.135 *	0.081
PTSD_3_→PSS_4_	0.139	0.282	−0.012	0.018	−0.160 *	0.072	−0.188 *	0.119
Covariance Coefficients								
PTSD_1_<->PSS_1_	−0.081	0.129	0.005	0.120	−0.405 **	0.136	−0.223	0.243
PTSD_2_<->PSS_2_	−0.292	0.163	0.072	0.143	−0.455 ***	0.126	−0.267	0.325
PTSD_3_<->PSS_3_	0.008	0.197	0.437 *	0.197	−0.081	0.124	−0.327	0.249
PTSD_4_<->PSS_4_	−0.909	0.657	−0.387 *	0.174	−0.251 *	0.109	−0.173	0.268
RI_PTSD_RI_PSS_	−0.346 **	0.121	−0.303 **	0.104	−0.144	0.254	0.001	0.229

Note: PSS = perceived social support. Est. = standardized parameter estimate. RI_x_RI_y_ = covariance between PTSD random intercept and social support random intercept. * *p* < 0.05. ** *p* < 0.01. *** *p* < 0.001.

## Data Availability

Data and study analysis codes are available from the authors upon reasonable request.

## Supplementary Materials

The following supporting information can be downloaded at: https://www.mdpi.com/article/10.3390/bs14040284/s1, Supplemental Table S1. Sample age and gender by ethnicity. Supplemental Table S2. Correlations between PTSD and social support at each time point.
