# Supplementary material for: Sources of Social Support and Trauma Recovery: Evidence for Bidirectional Associations from a Recently Trauma-Exposed Community Sample
Source: Behav Sci (Basel). 2024 Mar 29;14(4):284. doi: 10.3390/bs14040284 (PMC11047467; doi:10.3390/bs14040284)
Supplement: Supplementary file 1 [file behavsci-14-00284-s001.zip › behavsci-2855331-supplementary.pdf]

Supplemental Table S1. Sample age and gender by ethnicity.

Ethnicity	*n* (%)	*n* self-identified women/ % subsample	Age, *M* years *(SD)*
Aboriginal	6 (4.0)	6 (100.0)	41.05 (13.81)
Black	21 (13.9)	12 (57.1)	34.76 (11.84)
South, West, South East, East Asian	30 (19.8)	23 (76.7)	30.7 (12.73)
White	72 (47.7)	50 (69.4)	39.52 (14.18)
Mixed or Other Ethnicity	18 (11.8)	13 (72.2)	40.20 (15.40)

Supplemental Table S2. Correlations between PTSD and social support at each time point

	2.	3.	4.	5.	6.	7.	8.	9.	10.	11.	12.	13.	14.	15.	16.	17.	18.	19.	20.
1. T1 CAPS	.75**	.66**	.62**	-.28**	-.29*	-.30*	-.21*	-.19*	-.22*	-.21*	-.17	-.31**	-.29**	-.29**	-.17	-.27*	-.04	-.29	-.07
2. T2 CAPS	-	.82**	.82**	-.21*	-.29*	-.32*	-.16	-.16	-.19*	-.23*	-.13*	-.22*	-.32**	-.36**	-.16	-.29*	-.12	-.15	-.13
3. T3 CAPS		-	.85**	-.18	-.28*	-.28*	-.16	-.17	-.17	-.17	-.11	-.15	-.32**	-.32**	-.19	-.19	-.14	-.28	-.10
4. T4 CAPS			-	-.25*	-.39*	-.42*	-.29*	-.17	-.25**	-.28**	-.24**	-.24**	-.43**	-.44**	-.29**	-.35*	-.25	-.36*	-.17
5. T1 PSRS total				-	.76**	.75**	.60**	.86**	.68**	.65**	.52**	.85**	.59**	.57**	.43**	.83**	.50**	.56**	.45**
6. T2 PSRS total					-	.94**	.69**	.65**	.86**	.81**	.67**	.65**	.85**	.78**	.50**	.49**	.59**	.67**	.28
7. T3 PSRS total						-	.69**	.61**	.80**	.85**	.66**	.64**	.80**	.86**	.49**	.50**	.61**	.63**	.35*
8. T4 PSRS total							-	.57**	.60**	.61**	.87**	.41**	.55**	.55**	.83**	.54**	.34*	.36*	.63**
9. T1 PSRS Relatives								-	.72**	.69**	.64**	.50**	.38**	.34**	.29**	.55**	.36*	.31	.34*

10. T2 PSRS Relatives						-	.94**	.74**	.48**	.52**	.46**	.30**	.26	.16	.25	.04
11. T3 PSRS Relatives							-	.76**	.45**	.48**	.51**	.28**	.20	.20	.11	.10
12. T4 PSRS Relatives								-	.26**	.41**	.39**	.50**	.28	.09	.06	.27
13. T1 PSRS Friends									-	.63**	.62**	.43**	.69**	.40**	.54**	.33*
14. T2 PSRS Friends										-	.94**	.55**	.49**	.51**	.66**	.28
15. T3 PSRS Friends											-	.54**	.51**	.57**	.61**	.39*
16. T4 PSRS Friends												-	.37*	.24	.35*	.39**
17. T1 PSRS Intimate Partner													-	.59* *	.68**	.70**
18. T2 PSRS Intimate Partner														-	.07**	.68**
19. T3 PSRS Intimate Partner															-	.65**
20. T4 PSRS Intimate Partner																-

Note. CAPS = Clinician-Administered PTSD Scale for DSM-IV [33]; PSRS = Provision of Social Relations Scale [17]; T1 = First assessment; T2 = Second assessment; T3 = Third assessment; T4 = Fourth assessment. * $p < .05$, ** $p < .01$.
